# Impact of three exogenous phosphorus-solubilizing bacteria on zinc and selenium contents and rhizosphere soil nutrients of Longjing and Huangjinya tea plants

**DOI:** 10.3389/fmicb.2024.1413538

**Published:** 2024-06-26

**Authors:** JinMei Guo, ShuQing Zhang, JianFeng Li

**Affiliations:** ^1^School of Geography and Resources, Guizhou Education University, Guiyang, China; ^2^Institute of Soil and Environment Bioremediation in Karst Habitats, Guizhou Education University, Guiyang, China; ^3^Key Laboratory of Biological Resources Exploitation and Utilization in Colleges and Universities of Guizhou Province, Guiyang, China

**Keywords:** phosphorus-solubilizing bacteria, tea plants, rhizosphere soil nutrients, available Se, available Zn

## Abstract

Phosphate-solubilizing bacteria (PSB) enhance plant phosphorus utilization through their ability to dissolve phosphorus. To address the low utilization of nitrogen, phosphorus, potassium, zinc, and selenium by tea plants in acidic, selenium-rich soils, the study aimed to investigate the impact of exogenous PSB on soil nutrients and the absorption of zinc and selenium by tea plants. Following the inoculation of potted Longjing and Huangjinya varieties with exogenous phosphorus-solubilizing bacteria, we determined the concentrations of AN, AP, AK, Zn, and Se in their rhizosphere soil, in addition to the Zn and Se contents in their aboveground and belowground parts. The results show that after respective treatment with the three PSB, the concentration of available P in the tea plant rhizosphere soil significantly increased, with PMS08 having the most pronounced effect.After the same treatment, In the rhizosphere soil of Longjing tea plants, the AN content increased by 26.47%, 18.41%, and 7.51%, respectively, relative to the control, while the AK content decreased in the rhizosphere soil of Huangjinya tea plants. Inoculation with the three PSB resulted in a greater content of available Se in both the aboveground and belowground parts of the two tea plants. After inoculation with PMS20, the available Zn content of the belowground parts of Longjing and Huangjinya tea plants respectively decreased by 13.42% and 15.69% in comparison with the control. Additionally, after inoculating Longjing tea plants with PSt09 and Huangjinya tea plants with PMS08, the content of available Zn in their belowground parts significantly decreased by 9.22% and 35.74%, respectively. Evidently, the inoculation with the three phosphorus-solubilizing bacteria is beneficial for the uptake of available P by tea plants, promoting the utilization and accumulation of available Se. However, the content of AN or AK in rhizosphere soil varies between different tea plant varieties inoculated with the same kind of phosphorus-solubilizing bacteria. Moreover, the content of available Zn in tea plants also differs, highlighting the need to further investigate the differential effects of phosphorus-solubilizing bacteria on different plant varieties.

## 1 Introduction

Phosphate-solubilizing bacteria (PSB) play a crucial role in soil biogeochemical cycles due to their ability to solubilize inorganic phosphorus and mineralize organic phosphorus, which are among the most important physiological functions of soil bacteria (Billah et al., 2019). The low-molecular-weight organic acids synthesized by PSB can chelate cations that bind to phosphorus, thus transforming insoluble inorganic phosphorus into a soluble form; and PSB synthesize different phosphatases to catalyze the hydrolysis of phosphorus esters and to promote the mineralization of organophosphorus forms (Dastager and Damare, 2013). Not only can PSB improve the utilization rate of phosphorus in plants, as well as plant growth and development conditions, including soil structure (Xiao et al., 2013; Alori et al., 2017), but they also promote the adsorption of trace elements such as zinc, copper and calcium by roots, and enhance the absorption efficiency of soil nutrients by plants. In this way, PSB are beneficial for the growth and development of many plants, improve their quality and yield (Elhaissoufi et al., 2022). The distribution of phosphorus-solubilizing microorganisms in soil has a strong rhizosphere effect; that is, compared with non-rhizosphere soil, PSB occur at higher concentrations in rhizosphere soil (Tang et al., 2022), while the distribution of PSB differs among same crops’ rhizosphere (Guo et al., 2023) and also under differing conditions or soil types (Fiore-Donno et al., 2022).

In most soils, nitrogen, phosphorus and potassium are key indicators of soil fertility (Manzoor et al., 2017; Yunxiang et al., 2021). Hence, their content and regulation in agricultural soil have always received due attention from agricultural management departments. However, these nutrient elements in tea garden soil are usually insufficient meet the growth needs of tea trees (Liu et al., 2021). Further, the unreasonable application of chemical fertilizers typically introduces a series of soil problems (Yan et al., 2018). Research by Kumawat et al. (2024) indicates that multifunctional PGP activities mitigate agricultural stress. The use of microbiological systems to ameliorate the agricultural production in a sustainable and eco-friendly way is widespread accepted as a future key-technology (Kumawat et al., 2022).

Nitrogen (N), phosphorus (P), potassium (K), zinc (Zn), and selenium (Se) all play an important role in the growth and development of tea plants. Nitrogen and phosphorus are involved in the metabolic process of plants in many forms, and are essential for their growth and development, and thus figure prominently in yield and quality formation of crops (Deressa et al., 2012; Weeks and Hettiarachchi, 2019). Potassium plays a key role in the Hill reaction, primarily associated with the generation of NADPH and ATP, and in the Calvin–Benson cycle, it is essential for CO_2_ fixation and the production and transport of sugars, while also regulating the synthesis of biological macromolecules and promoting sugar transport (Wakeel and Magen, 2017). Zinc can affect the growth of tea plants, the development of plant reproductive organs, the content of mineral elements in mature leaves, and plant resistance to stress (Gupta et al., 2016). Although not necessary for plant growth, selenium absorption can promote it with reproduction in addition to enhancing stress resistance responses (Cabot et al., 2019).

To address the problem of their low-utilization rate of nitrogen, phosphorus, potassium, and zinc and selenium in acidic selenium-rich soil, Longjing and Huangjinya tea plants were inoculated in this study with three exogenous high-efficiency PSB. The effects of different PSB and the availability of nutrients in the rhizosphere of the two different tea varieties were analyzed. Building on these results, the relevance of different PSB for the absorption of zinc and selenium by both tea varieties was discussed, with a view to improving the soil nutrient cycle and promoting the nutrient absorption and dry matter accumulation of tea trees. Our study’s findings provide a scientific basis for enhancing the quality of tea and increasing its yield.

## 2 Materials and methods

### 2.1 Test soil

The soil sampling site was in a natural woodland of Anping Village, Fengsan Town, Kaiyang County, in central Guizhou (106°59°48.77″ N, 27°12°10.04″ E), at 1086 m above sea level. In this area, the forest coverage is about 54%, the annual average temperature is about 10.6–15.2°C, and the soil is a red brown type with a pH value of 4.36. The basic nutrient data are presented in Table 1.

**Table 1:** Basic data of potting soil.

Soil nutrient	Available N (mg kg^−1^)	Available P (mg kg^−1^)	Available K (mg kg^−1^)	Available Zn (mg kg^−1^)	Available Se (mg kg^−1^)	Organic matter (g kg^−1^)	Total Se (g kg^−1^)
Content	66.16 ± 2.23	44.09 ± 0.98	75.60 ± 3.44	3.47 ± 0.11	0.06 ± 0.01	28.41 ± 0.96	1.44 ± 0.03

### 2.2 Bacterial liquid strains tested

The test strains PMS08, PMS20, and PSt09 were isolated from the roots of wild tea plants in the Anping Village of Kaiyang County, in Guiyang City (Guizhou Province, China), these provided by the Institute of Soil and Environmental Bioremediation in Karst Habitats. Through 16S sequencing, PMS08 and PMS20 were identified as *Paraburkholderia fungorum* capable of organic phosphorus solubilization, while PSt09 was identified as *Enterobacter wuhouensis* capable of both organic and inorganic phosphorus solubilization. After their isolation and purification, the strains were put into LB liquid medium. After shaking each at 150 r/min until an OD600nm between 0.5 and 1 was reached, the bacterial solution was centrifuged at 4,000 r/min for 10 min. After discarding the supernatant, sterile water was added. When the OD value reached 0.8, the concentration of phosphate-solubilizing bacteria was determined to be 8 × 10^8^ CFU mL^−1^, which constituted the desired bacterial solution.

### 2.3 Test plants and treatments

Seedlings of the Longjing and Huangjinya tea varieties with a height of 1 meter were provided by the Meitan Yongxing Yongshun Seedling Planting Co., Ltd. After removing any impurities from the above-mentioned test soil, it was added to a planting frame (80 cm in length, 40 cm in width, and 23 cm in height), 4 plants were transplanted into each frame, and each bacterial treatment group and control group were repeated 3 times. After the tea seedlings resumed growth and new leaves displayed, the treatment group slowly poured 50 mL of the tested bacteria solution into the roots of tea seedlings until the soil was fully absorbed, while the control group was treated with sterile water. After 60 days, the rhizosphere soil, aboveground part and underground part of the plant in the treatment group and the control group were collected, respectively.

### 2.4 Elements’ determination

The respective content of available N (AN), available P (AP), and available K (AK) in soil was determined according to the *Soil Agrochemical Analysis* (3rd ed.) (Bao, 1997), which was edited by Nanjing Agricultural University. Of the AN, its content of nitrate nitrogen was determined by phenol diacid colorimetry; that of ammonium nitrogen was determined by 2 mol L^−1^KCI extraction distillation method. To determine the AP, the 0.05 mol L^−1^ HCl-0.025 mol L^−1^ (1/2H_2_SO_4_) method was used. The AK was determined via ammonium acetate (NH_4_OAc) extraction and flame photometry.

The contents of soil available Se and tea plant Se were quantified by following the method described by Gou et al. (2013).

According to the “GB 7680-87 determination of available zinc in forest soil” and the “determination of total silicon, iron, aluminum, calcium, magnesium, potassium, sodium, phosphorus, sulfur, manganese, copper and zinc in forest plants and forest litter,” the contents of soil available Zn and tea plant Zn were determined.

### 2.5 Data processing

The standard error and average value of three replicates of the test data were calculated. Their one-way ANOVA (analysis of variance) and the LSD multiple comparison method were implemented using SPSS 23.0 software. Origin 2021 software was used to draw the graphs.

## 3 Results

### 3.1 Effects of PSB on the AN in tea rhizosphere soil

**Figure 1:**
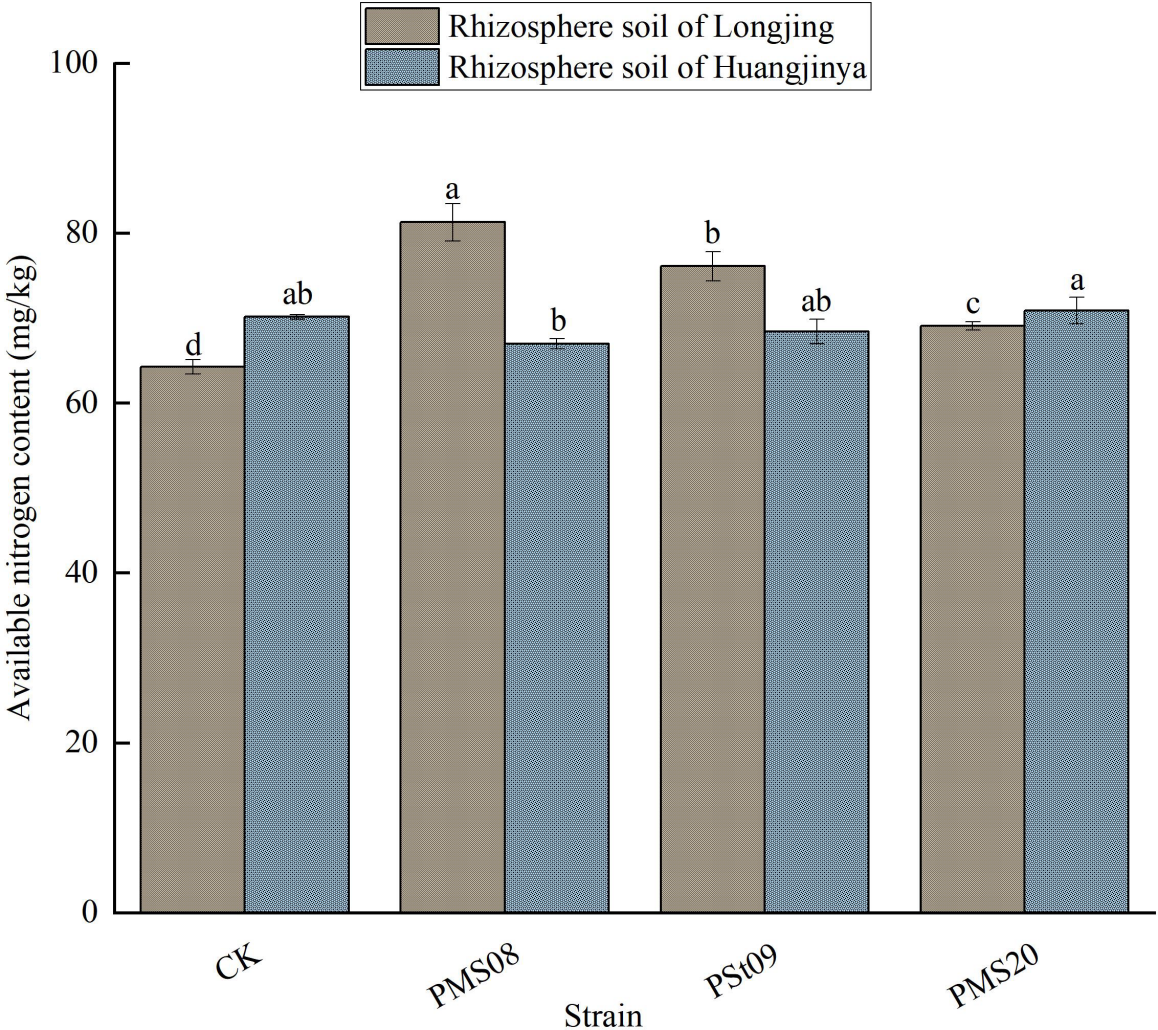
Effects of PSB on AN content in tea plants rhizosphere soil. Different lowercase letters indicate significant differences (*p* < 0.05). The same for Figures 2–9 below.

As Figure 1 shows, after their inoculation with PMS08, PSt09, and PMS20 strains, the Longjing tea plants’ rhizosphere soil had a 26.47, 18.41, and 7.51% greater AN content than the control, and these difference were significant (*p* < 0.05). After the PMS08 treatment, the soil AN content was 81.30 mg/kg, which significantly exceeded that of the other two strain treatments. However, the AN content of rhizosphere soil was similar between Huangjinya varieties and the control group without any exogenous PSB. After inoculation of PMS08 and PMS20, the content of AN was significantly higher in the rhizosphere soil of Longjing varieties than Huangjinya varieties. After inoculation of PSt09, the content of AN in rhizosphere soil of Longjing tea plants was higher than that Huangjinya, but this difference was not significant.

These results showed that inoculation of PMS08 and PMS20 with soluble organic phosphorus and PSt09 with soluble organic and inorganic P had positive effects on the AN content of Longjing tea plant soil.

### 3.2 Effects of PSB on the AP in tea rhizosphere soil

**Figure 2:**
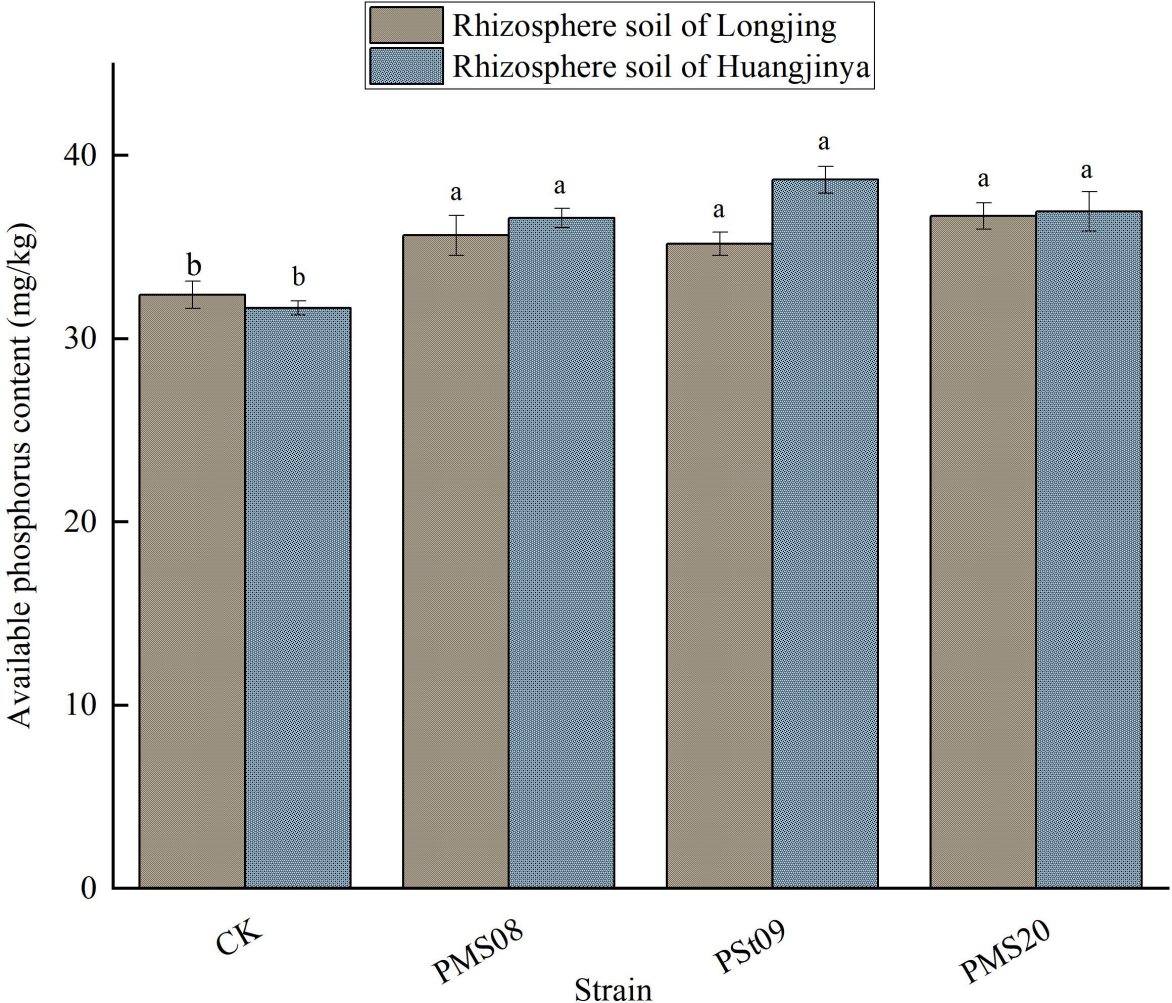
Effects of PSB on the content of AP in tea rhizosphere soil.

Inoculation with PSB could significantly increase the content of AP in the rhizosphere soil of Longjing and Huangjinya tea plants (*p* < 0.05; Figure 2). The AP content of Longjing tea rhizosphere soil inoculated with PMS08, PSt09, and PMS20 was 10.03, 8.58, and 13.30% higher than that of the control, respectively; the corresponding relative increases were 15.54, 22.08, and 16.67% for Huangjinya. There was no significant difference in the AP content between the two strains PMS08, PMS20 and PSt09, which could dissolve organic P and inorganic P, and likewise between Longjing and Huangjinya treatment groups. The three exogenous strains were highly efficient PSB, which could improve the content of AP in the rhizosphere soil of these two tea varieties.

### 3.3 Effects of PSB on the AK in tea rhizosphere soil

**Figure 3:**
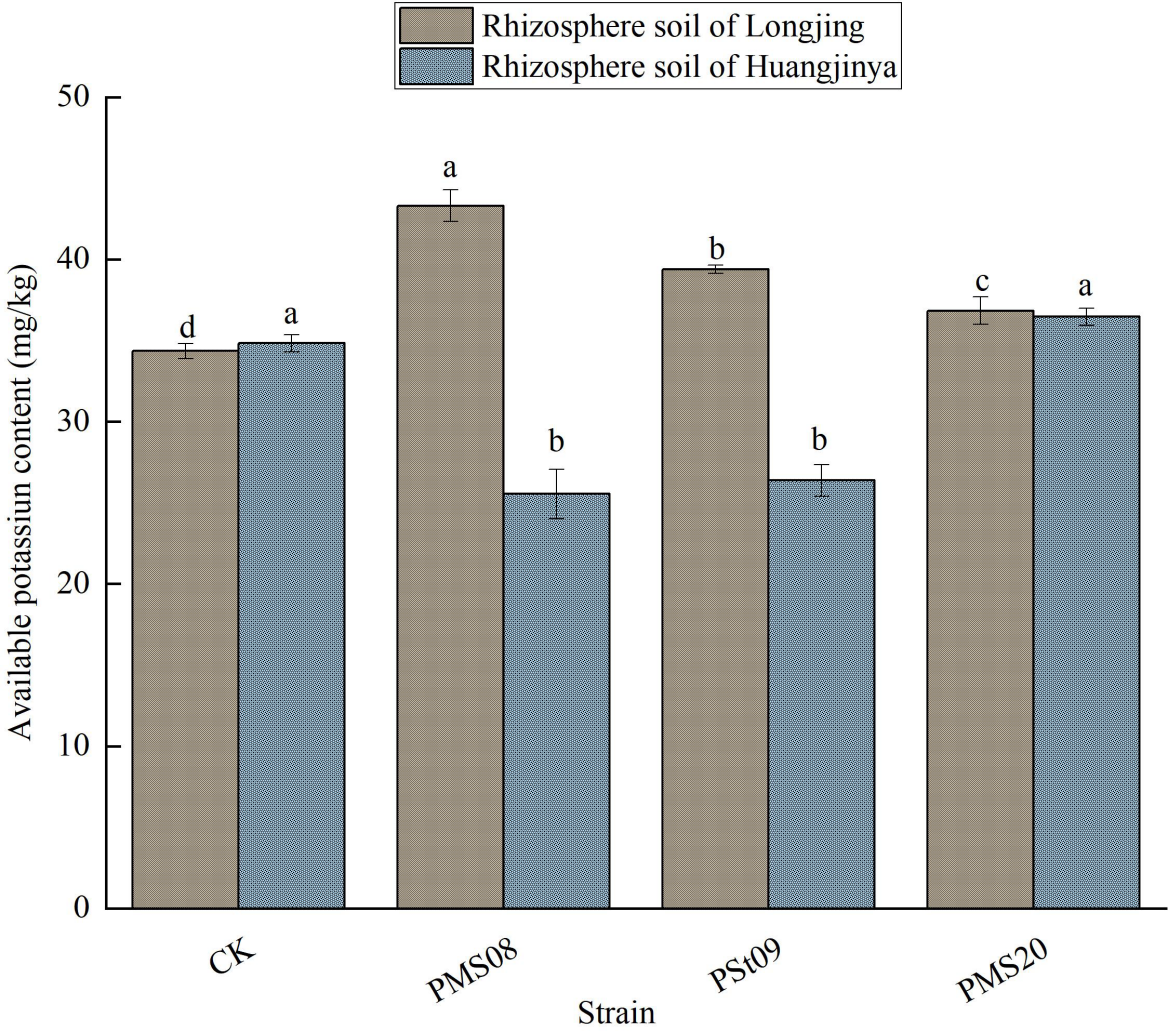
Effects of PSB on the content of AK in tea rhizosphere soil.

As seen in Figure 3, all the three PSB could significantly increase the content of AK in the rhizosphere soil of Longjing tea seedlings, especially when treated with the PMS08 strain, this being 26.05% higher *vis-à-vis* the control group (*p* < 0.05). After treating the Huangjinya tea seedlings with each strain, the AK content of their rhizosphere soil was unlike that for Longjing tea seedlings: only inoculation with PMS20 strain led to a slightly higher AK content than the control. Hence, the same PSB can differentially affect the content of AK in the rhizosphere soil of different tea varieties.

### 3.4 Effects of PSB on the available Zn in tea rhizosphere soil

**Figure 4:**
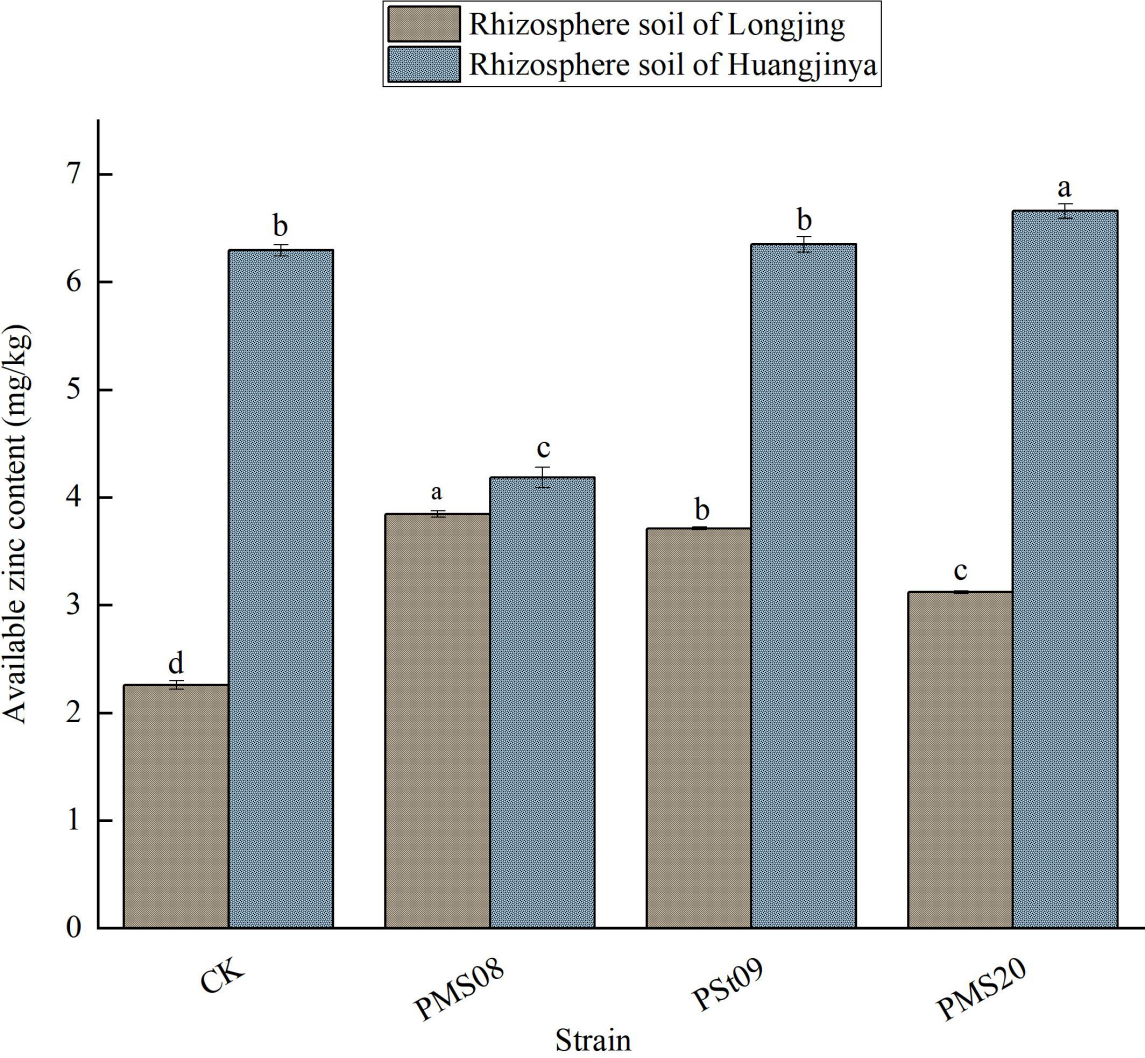
Effect of PSB on the content of available Zn in rhizosphere soil.

As Figure 4 shows, all the three PSB could significantly increase the content of available Zn in the rhizosphere soil of Longjing tea seedlings. After inoculating them with PMS08, the available Zn content increased by 70.25% (*p* < 0.05). Evidently, however, the same strain of PSB did not affect both tea varieties in the same way. After administering PMS20, the content of available Zn in the rhizosphere soil of Longjing and Huangjinya tea plant plants increased by 38.13 and 5.79%, respectively. After inoculation with PSt09, for Longjing tea rhizosphere soil, its available Zn content increased by 64.39%, being significantly greater than the control, whereas the increase amounted to just 0.91% in Huangjinya tea rhizosphere soil, being similar to the control. To sum up, the content of available Zn in Longjing tea rhizosphere soil increased significantly after inoculation with the three kinds of PSB, whose effects on Huangjinya tea rhizosphere soil were different.

### 3.5 Effects of PSB on the available Se in tea rhizosphere soil

**Figure 5:**
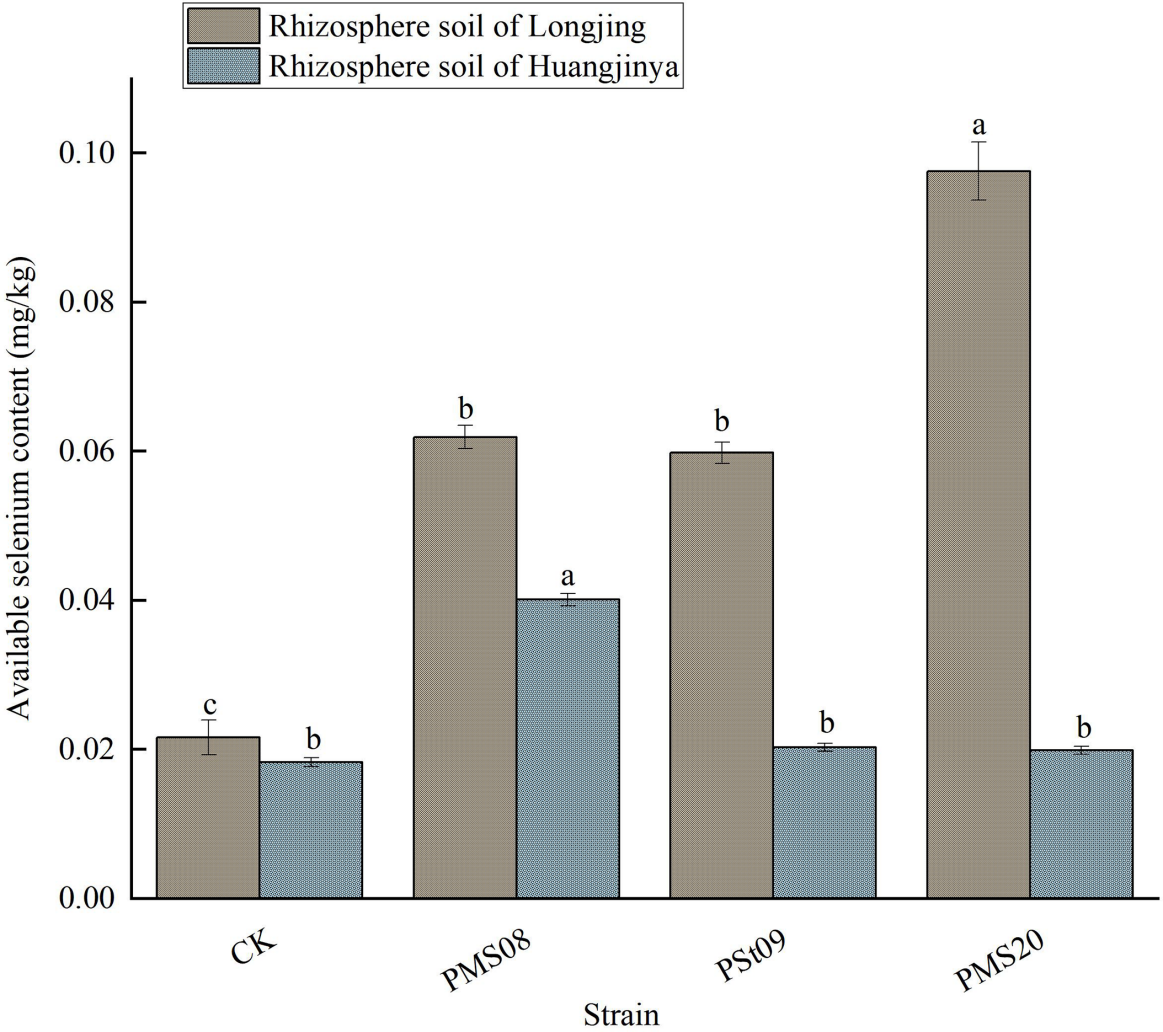
Effects of PSB on available Se content in tea rhizosphere soil.

After inoculating them with PMS08, the available Se content in the rhizosphere soil of Longjing and Huangjinya tea plants increased significantly (Figure 5), respectively increasing by 186.57 and 119.13% *vis-à-vis* the control. After applying PMS20 and PSt09, the content of available Se in Longjing tea rhizosphere soil increased significantly, to 176.85 and 351.85% that of the control, respectively, while for Huangjinya the corresponding increases were lower (10.93 and 8.74%). Notably, PMS08 had significant positive effects on the available Se in rhizosphere soil of both varieties, while all three kinds of PSB were capable of significantly improving the content of available Se in the rhizosphere soil of Longjing tea plants.

### 3.6 Effects of PSB on the zinc content of Longjing tea plants

**Figure 6:**
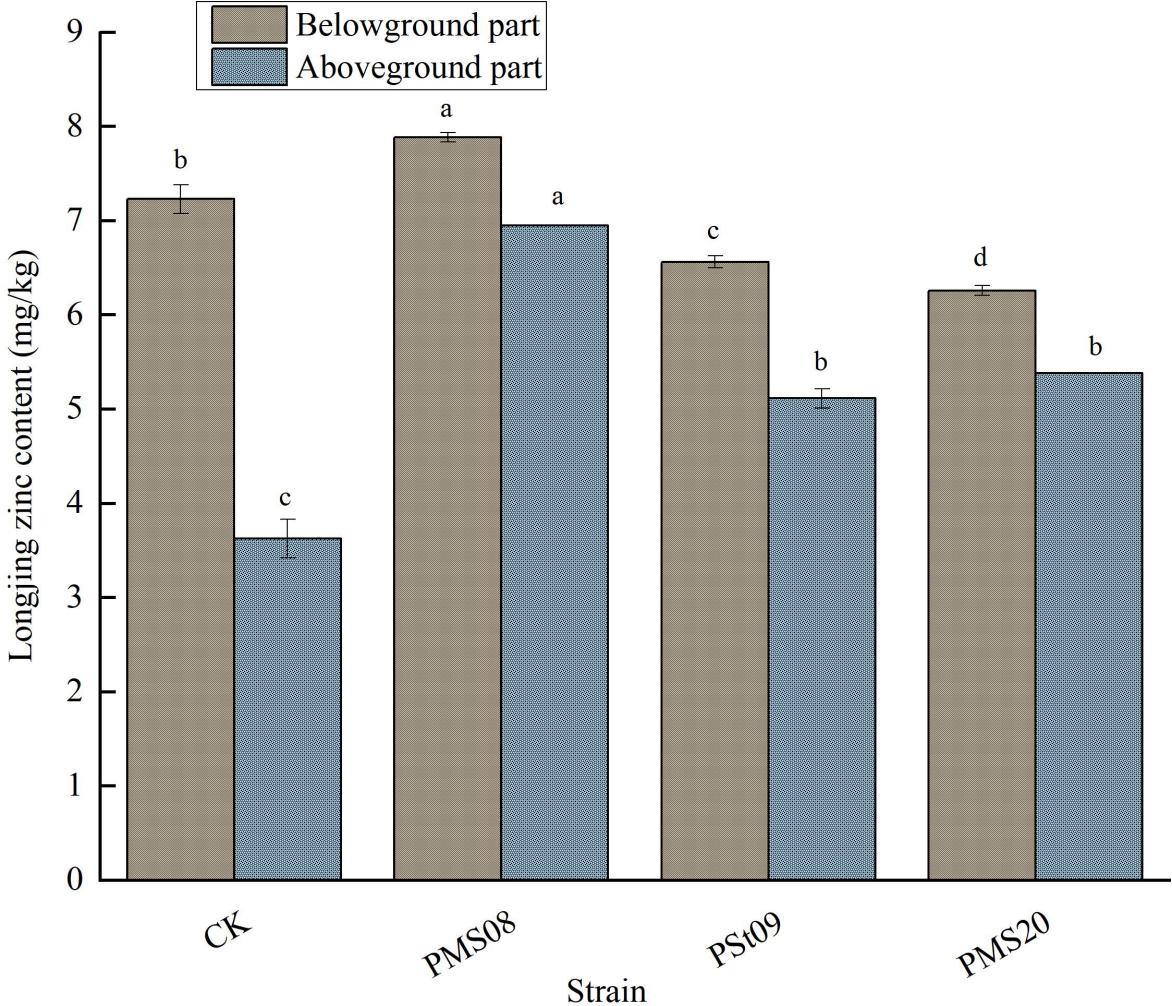
Effect of PSB on Zn content of Longjing tea plants.

As illustrated in Figure 6, the Zn content of the aboveground and belowground parts of Longjing tea plants differed after inoculation with PSB. Under each strain treatment, the aboveground Zn content in their aboveground part significantly increased by 91.59, 48.42, and 40.98%, respectively, relative to the control (*p* < 0.05). When treated with PMS08, the Zn content belowground increased significantly, by 9.10% (*p* < 0.05). Thus, it can be seen that PSB can significantly promote the accumulation of Zn in the aboveground part of Longjing seedlings, implying the strain promoted greater transport of soil available Zn to their stems and leaves.

### 3.7 Effects of PSB on the Zn content of Huangjinya tea plants

**Figure 7:**
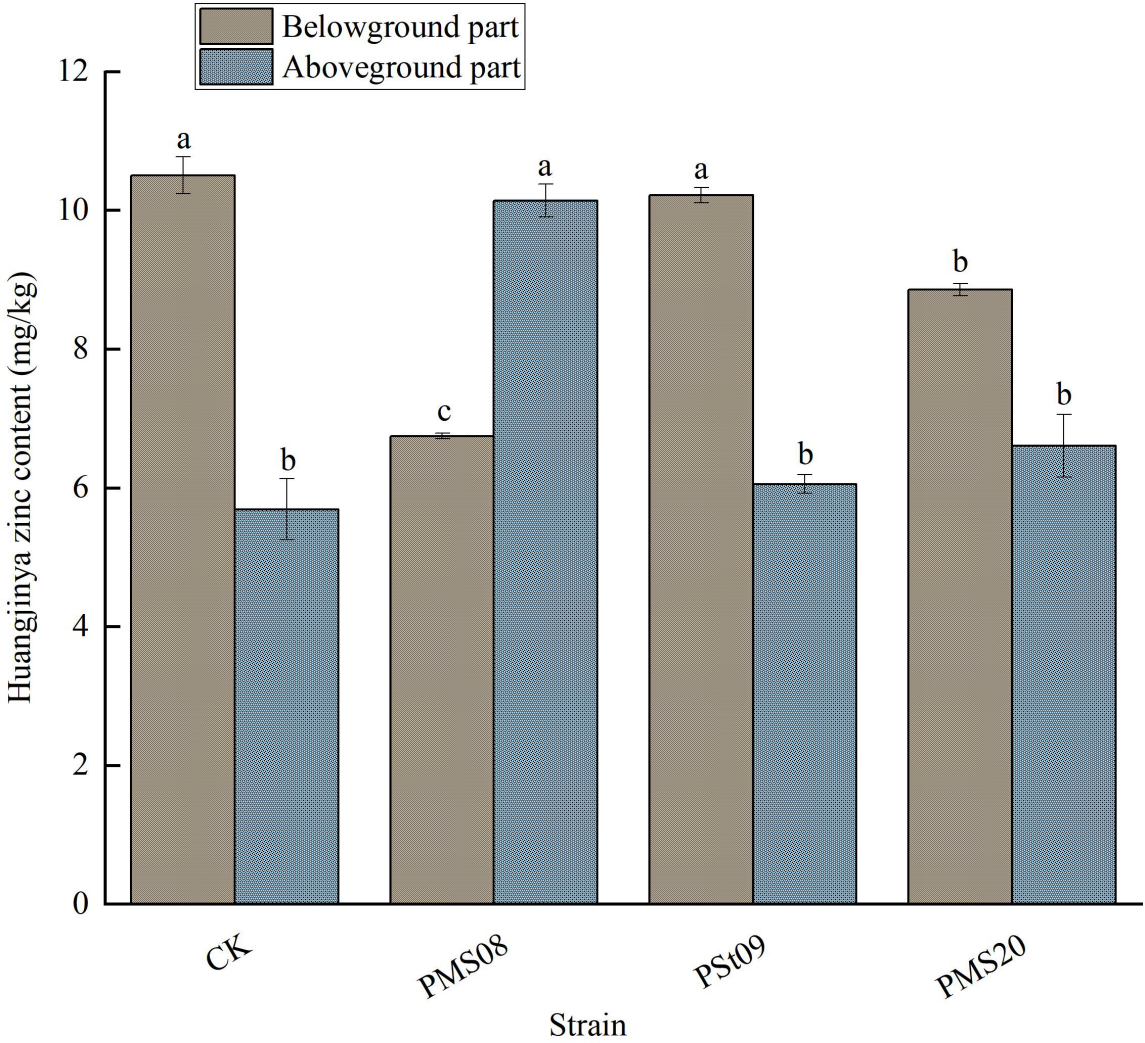
Effect of PSB on Zn content of Huangjinya tea plant plants.

As Figure 7 shows, the Zn content of the aboveground and belowground parts of Huangjinya tea plants was affected differently by inoculation with PSB. After applying each strain to those plants, to varying degrees their Zn content decreased belowground and increased aboveground. In the latter part the Zn content was 78.20% higher than that of the control yet 35.74% lower in the former part after the PMS08 inoculation. These results indicated PSB played a positive role in the accumulation of Zn in the aboveground part of Huangjinya tea seedlings, with the PMS08 strain able to promote the upward transport of Zn in them.

### 3.8 Effects of PSB on the Se content of Longjing tea plants

**Figure 8:**
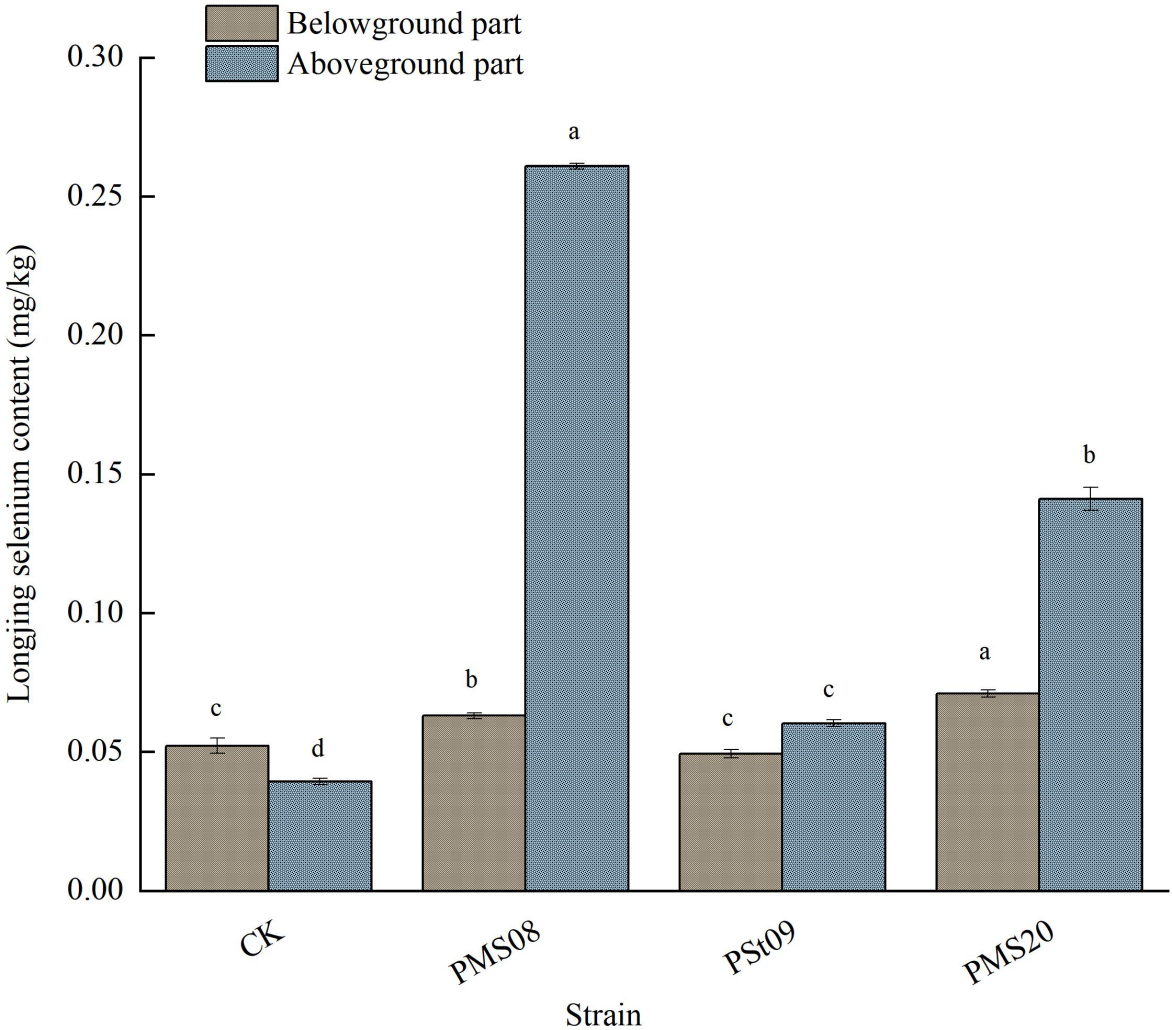
Effect of PSB on Se content of Longjing tea plants.

The Se content in the aboveground and belowground parts of Longjing tea plants increased to varying degrees after inoculation with PSB (Figure 8). Under the PMS08, PMS20, and PSt09 strain treatments, the Se content in the aboveground part of Longjing tea plants significantly increased by 563.87, 259.03, and 53.44% compared with the control, respectively, reaching 0.26 mg/kg. The belowground part’s Se content increased significantly, by 20.69 and 36.02% relative to the control, under the PMS08 and PMS20 strain treatments, respectively. Evidently, the two organophosphorus-solubilizing strains were able to promote the absorption and accumulation of selenium in Longjing plants, and their inoculation with PSB contributed to augmenting their Se content, especially aboveground.

### 3.9 Effects of PSB on the available Se content of Huangjinya tea plants

**Figure 9:**
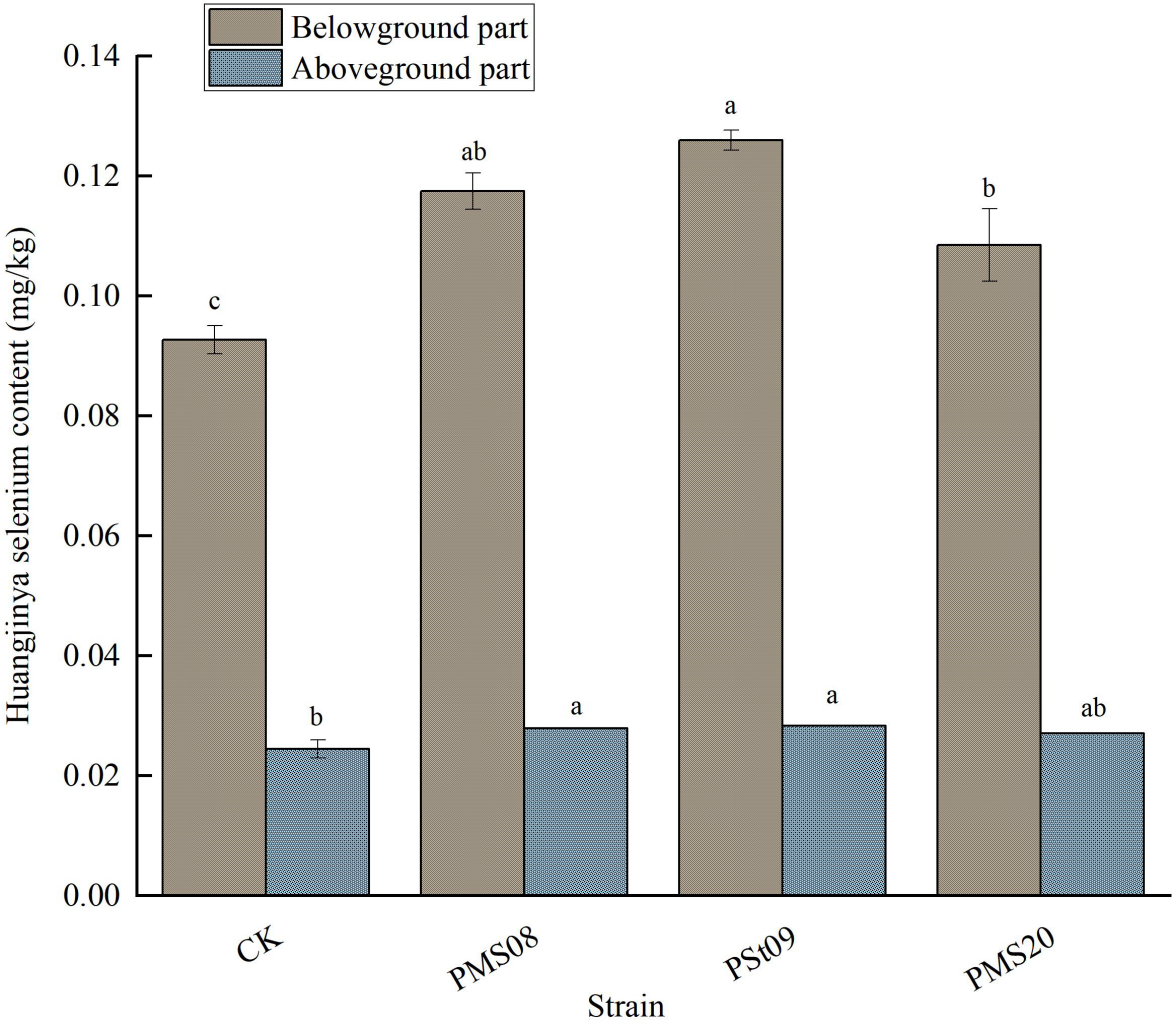
Effect of PSB on Se content of Huangjinya tea plants.

As Figure 9 shows, compared with the control, the available Se content of Huangjinya plants inoculated with the three PSB was 13.87, 10.61, and 15.51% higher in their aboveground part, and 26.75, 17.04, and 35.92% higher in their belowground part. All these differences were significant (*p* < 0.05). Among them, the content of available Se peaked in the belowground part of Huangjinya tea plants treated with PSt09, and the increase in available selenium was greater belowground than aboveground. These results indicated that the accumulation of available Se in tea plants was a bottom-up process, and that inoculation with PSB could, to a certain extent, promote the vertical transport of available Se from roots up to the stem and leaf tissues.

### 3.10 Correlation analysis of AN, AP, AK, Zn, Se contents in the rhizosphere soil and parts of tea plants

**Figure 10:**
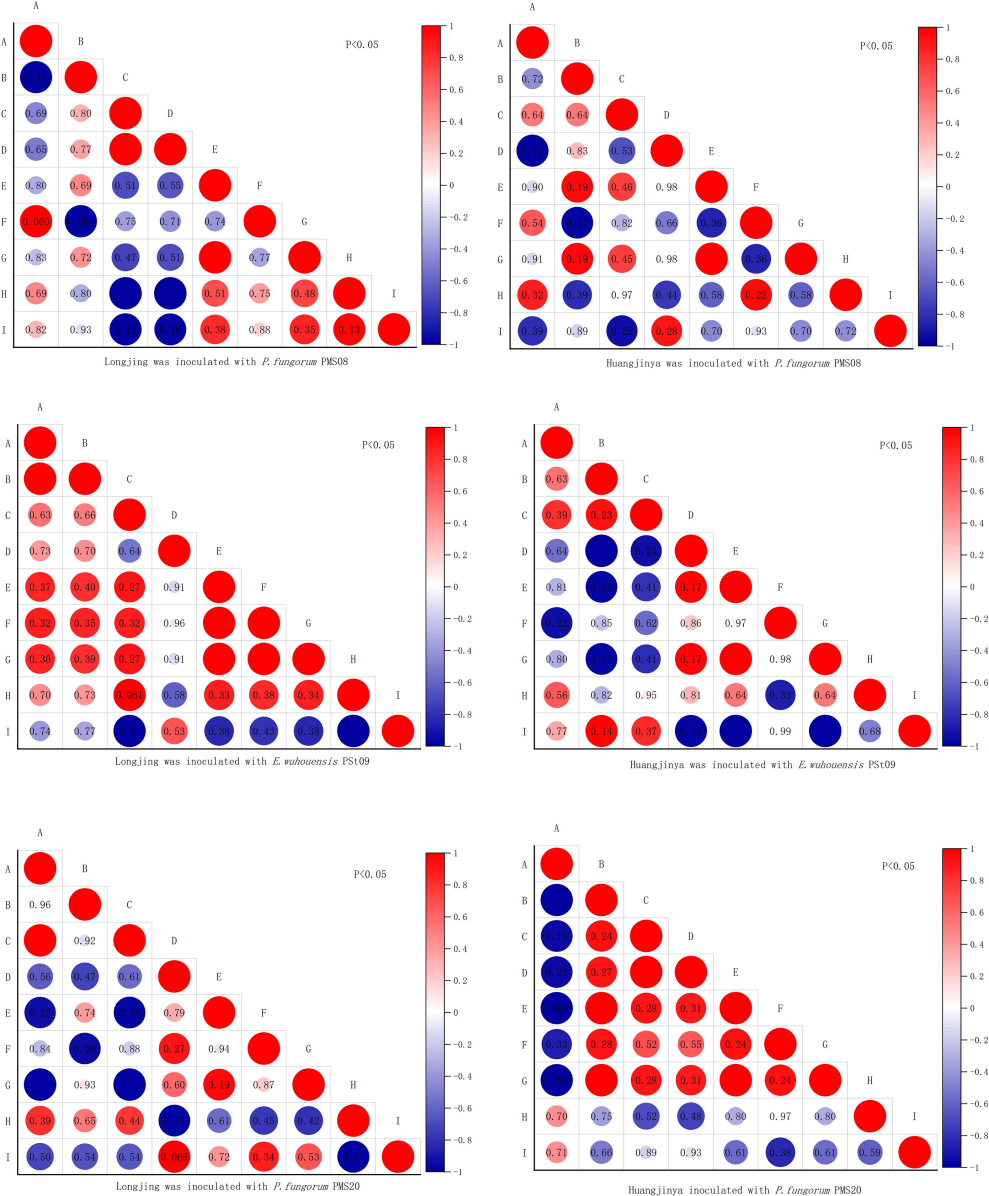
Correlation analysis of AN, AP, AK, Zn and Se contents in tea rhizosphere soil and tea plants. (A–I) Respectively denotes the determination index: belowground Zn, aboveground Zn, belowground Se, aboveground Se, available Zn in rhizosphere soil, available Se in rhizosphere soil, AN in rhizosphere soil, AP in rhizosphere soil, AK in rhizosphere soil.

As Figure 10 shows, at 60 days post-inoculation with PMS08, there was a positive correlation among AP, Zn, Se and AN in Longjing tea rhizosphere soil. Similarly, there was a positive correlation between AK and Zn, AN, AP in rhizosphere soil. Belowground Se and aboveground Se were significantly positively correlated. For Huangjinya tea plants, there was a significant positive correlation between Zn and AN in their rhizosphere soil.

After applying the PSt09 strain, there was a significant correlation between aboveground and belowground Zn of Longjing tea plants, with significant positive correlations in the rhizosphere soil between its AN and available Zn or Se. The positive correlation also existed between the contents of Zn and Se in Longjing plant, and between Zn and Se in rhizosphere soil. AN and AP in rhizosphere soil also had a positive correlation. The rhizosphere soil’s AN and available Zn were significantly positively correlated, and both likewise with aboveground Zn.

After inoculating with PMS20, there positive correlations between rhizosphere soil AP and aboveground Zn, belowground Zn, Se content, rhizosphere soil AK and rhizosphere soil AN, and a significant positive correlation between belowground Zn and Se. The aboveground Zn of Huangjinya tea plants was positively correlated with their belowground Se, rhizosphere soil available Zn and Se, and significantly positively correlated with aboveground Se and rhizosphere soil AN. Furthermore, rhizosphere soil AN was significantly positively correlated with aboveground Zn and rhizosphere soil available Zn, while the Se content was significantly correlated between aboveground and belowground parts of these plants. However, their rhizosphere soil AP was not strongly correlated with the other elements.

After inoculation with different PSB, in Longjing’s rhizosphere soil the available Zn and AP were positively correlated. For Huangjinya, all tea plants had a positive correlation between available Zn and AN in their rhizosphere soil. The effects of the same PSB treatment on these two different tea varieties were not consistent in terms of their elemental content correlations.

## 4 Discussion

### 4.1 Effects of phosphorus-solubilizing bacteria on the contents of AN, AP and AK in soil

Soil available nitrogen (AN), available phosphorus (AP), and available potassium (AK) refer to the available nutrients that can be directly absorbed and utilized by plants. Due to the influence of human activities, topographic factors, and soil physical and chemical properties, among other factors, AN, AP, and AK often exhibit strong spatial variability (Zhu et al., 2021). The P-solubilizing ability of phosphorus-solubilizing bacteria is influenced by many environmental factors, such as soil temperature, salinity, pH, and dissolved oxygen. The P-solubilizing mechanism of PSB in the crop rhizosphere can also differ across regions, soil types, and among various planting patterns (Wei et al., 2016; Zeng et al., 2016). In this experiment, the treatment of three strains of PBS was obviously beneficial to AP desorption, such that the content of AP in the rhizosphere soil of both Longjing and Huangjinya tea plants increased significantly.

Applying the PMS08, PMS20, PSt09 strains significantly increased the AN and AK in Longjing tea plants’ rhizosphere soil, results similar to those of Rawat et al. (2021) and Liu et al. (2004) who found that the application of P-solubilizing bacteria could significantly increase the content of plant dry matter, total P, and total N. This is roughly on par with PSB shown capable of increase the P and K contents of food crops (Gao et al., 2006), and our conclusion that PMS08 could be used as a high-quality resource for Longjing tea plants’ bacterial fertilizer.

However, the AK in their rhizosphere soil did decrease significantly after inoculation with PMS08 or PSt09, in contrast to the response of the Longjing variety. Hence the impact of PSB on AK in rhizosphere soil of different varieties of tea can differ considerably. In Huangjinya plants’ rhizosphere soil, AK decreased while AP increased significantly. Managers should consider to augmenting the application of potassium fertilizer when administering PSB to Huangjinya tea fields.

The kinetics of phosphorus in soil is a complex process, one jointly controlled by soil type, soil pH value, soil salinity, organic matter content, and metal oxides (Billah et al., 2019). Although PSB have been studied for many years, their practical development is limited with few widespread applications. It is generally believed that the main reason for that hindrance is the great diversity of and disparity across bacterial species with respect to their P-solubilizing ability when isolated from different soils (Liu et al., 2014). In our study, the contents of AN, AP, and AK in rhizosphere soil of different tea varieties inoculated with the same PSB trains were found to be quite different. The two tea germplasms used in this experiment originate from Zhejiang Province, China, albeit from locations over 100 kilometers apart, resulting in certain habitat differences. Importantly, Longjing belongs to common green tea, while Huangjinya is a yellow-variant optimized photosensitive green tea variety, which exhibits slightly lower stress resistance and relatively weaker cold and drought tolerance compared to common green tea. Under identical experimental conditions, the root systems of these two tea trees produce different secondary metabolites such as organic acids, affecting the efficiency of nutrient absorption and utilization in the soil. Furthermore, although the different growth-promoting bacteria strains used in this study all demonstrate phosphate solubilization capability, their proliferation, metabolism, and phosphate solubilization capacities vary in soil, thereby further widening the differences in nutrient content in the rhizosphere between different bacterial-tea tree combinations. The impact processes and mechanisms of PSB on different plant varieties may be more complex, necessitating further in-depth investigation in subsequent work.

### 4.2 Effects of PSB on the available Zn and Se in soil and their absorption by tea trees

In soil there is a complex coordination among P, Zn, and Se. Research has shown that P can improve desorption of selenate (Xue et al., 2020), and thus augment the availability of Se in soil. Many factors can affect the Se absorption by tea plants. Numerous studies have shown that the increase of P in soil will increase plant absorption of Se, and there is a synergistic effect between them (Xin et al., 2002). Zhao et al. (2013) found that the interaction between P and Se in soil was modulated by the latter’s content. Other work has shown that P can enhance plant transpiration and promote root growth, thereby indirectly promoting the passive absorption of Se (Yadav et al., 2023). This is consistent with our conclusion that applying PSB increases the content of available Se in soil and the aboveground and belowground parts of tea trees. At the same time, this study demonstrates that inoculation with the PMS08 strain could significantly promote the absorption of Se in aboveground and belowground parts of the two tea varieties.

It has been suggested that the addition of N or P, or both, would increase or decrease the content of Zn, Se, and other elements in crops (Yang et al., 2014; Hu et al., 2017; Grujcic et al., 2021). Furthermore, an increase in the availability of Zn or Se would in turn catalyze or inhibit the utilization of N and P in crops. In the present study, inoculation with three kinds of PSB clearly increased the content of both AN and AP in the rhizosphere soil of Longjing tea plants, but only that of AP in the rhizosphere soil of Huangjinya tea plants. In tandem, the content of available Se in aboveground and belowground parts of Longjing and Huangjinya tea plants also increased, yet the Zn content decreased significantly belowground for both tea varieties, after inoculating them with PMS20. However, only Zn of Longjing tea plants was affected inoculation with PSt09. In response to inoculation with PMS08, the content of Zn in the underground part of Huangjinya tea plants decreased significantly. These results are consistent with the above cited research findings, but not entirely so with the view put forward by Cakmak et al. (2010) and Li et al. (2017) whereby the Zn and Se contents of plant tissues are positively correlated with the available Zn and Se contents of soil. This discrepancy may be due to differences in the absorption and accumulation of AN, AP, and trace elements as regulated by different organs. In fact, the element distribution mechanisms that couple AN, AP and of Zn, Se and other trace elements in different plant organs is still unclear. Our study shows that even among different varieties of the same plant species, there may be more complex relationships between their tissue contents of Zn and Se *vis-à-vis* the amount of Zn and Se in soil.

## 5 Conclusion

For two varieties of green tea grown in selenium-rich soil, phosphate-solubilizing bacteria (PSB) isolated from the roots of wild tea trees can effectively solubilize phosphorus. Through the complex synergistic interaction between phosphorus and selenium, these bacteria enhance the availability of both elements in the soil, promoting tea tree growth and increasing the tea trees’ ability to absorb and translocate selenium. This is particularly applicable to phosphorus-deficient tea fields in the karst mountainous regions of Guizhou.

Even under the same environmental conditions, selenium-rich soil, and cultivation management practices, the nutrient dynamics in the rhizosphere can vary significantly among different combinations of tea tree varieties and PSB strains. For instance, all three PSB strains can increase the levels of available nitrogen (AN), available phosphorus (AP), available potassium (AK), and selenium (Se) in the rhizosphere soil of Longjing tea trees, but the extent of these increases varies among the strains. As a photosensitive tea variety with chlorosis variation, Huangjinya, when inoculated with the same PSB, shows different accumulations of nitrogen, phosphorus, and selenium in both the plant tissues and rhizosphere soil compared to the standard tea variety, Longjing. Consistently, PSB enhance the availability of phosphorus and selenium nutrients in the rhizosphere of both tea varieties, and as plant growth accelerates, the application of potassium fertilizer becomes necessary.

Regarding the available zinc (Zn) content, different tea tree varieties respond differently to various PSB strains. However, it is noteworthy that higher zinc content in the soil does not necessarily correlate with increased zinc bioavailability in plant tissues, indicating a complex relationship between soil zinc levels and plant uptake.

In conclusion, the efficacy of PSB inoculants in selenium-rich tea fields is closely linked to the specific tea tree varieties and soil nutrient conditions. Therefore, the development and application of PSB inoculants should be tailored to the particular soil conditions, crop varieties, and specific agricultural product traits required, such as the zinc and selenium content in tea leaves.

## Data availability statement

The original contributions presented in the study are included in the article/supplementary material, further inquiries can be directed to the corresponding author.

## Author contributions

JG: Data curation, Formal analysis, Writing – original draft. SZ: Funding acquisition, Writing – review & editing, Conceptualization. JL: Conceptualization, Writing – review & editing, Formal analysis.
